# HANDS: a tool for genome-wide discovery of subgenome**-**specific base-identity in polyploids

**DOI:** 10.1186/1471-2164-14-653

**Published:** 2013-09-24

**Authors:** Aziz Mithani, Eric J Belfield, Carly Brown, Caifu Jiang, Lindsey J Leach, Nicholas P Harberd

**Affiliations:** 1 Department of Plant Sciences, University of Oxford, South Parks Road, Oxford OX1 3RB, UK; 2 Department of Biology, Syed Babar Ali School of Science and Engineering, Lahore University of Management Sciences, D.H.A., Lahore 54792, Pakistan; 3 School of Biosciences, University of Birmingham, Edgbaston, Birmingham B15 2TT, UK

**Keywords:** Next-generation sequencing, RNA-seq, Polyploidy, Wheat, Base-identity

## Abstract

**Background:**

The analysis of polyploid genomes is problematic because homeologous subgenome sequences are closely related. This relatedness makes it difficult to assign individual sequences to the specific subgenome from which they are derived, and hinders the development of polyploid whole genome assemblies.

**Results:**

We here present a next-generation sequencing (NGS)-based approach for assignment of subgenome-specific base-identity at sites containing homeolog-specific polymorphisms (HSPs): ‘HSP base Assignment using NGS data through Diploid Similarity’ (HANDS). We show that HANDS correctly predicts subgenome-specific base-identity at >90% of assayed HSPs in the hexaploid bread wheat (*Triticum aestivum*) transcriptome, thus providing a substantial increase in accuracy versus previous methods for homeolog-specific base assignment.

**Conclusion:**

We conclude that HANDS enables rapid and accurate genome-wide discovery of homeolog-specific base-identity, a capability having multiple applications in polyploid genomics.

## Background

Polyploidy has played a key role in the evolution of many plants [1], and has important consequences with respect to transcriptional regulation and the silencing of genes [2-6]. However, polyploidy presents a particular problem with respect to genome analysis. Polyploid genomes consist of two or more homeologous subgenomes, and sequence relatedness between these subgenomes makes determining the homeoallelic identity of genes and gene transcripts a particular challenge [2].

**Figure 1:**
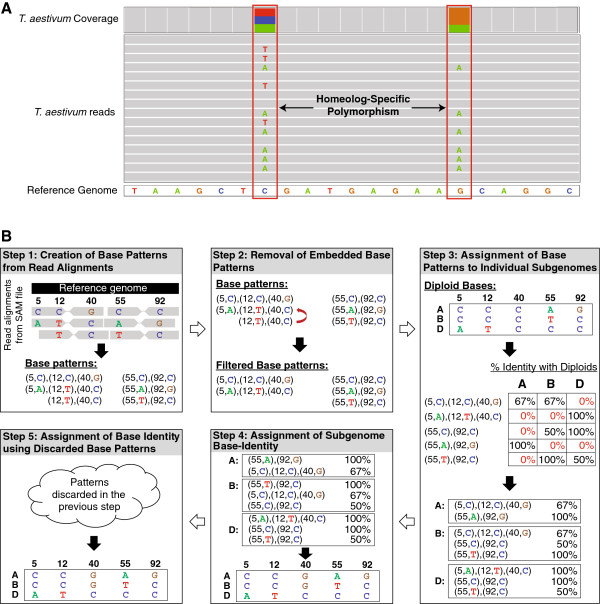
**Characterization of HSPs using HANDS. (A)** Illustration of Homeolog-Specific Polymorphisms (HSPs) in the *T. aestivum* genome. Bases that match the reference sequence are shown in grey and base substitutions (versus the reference sequence) are shown in other colors. Positions containing a HSP are highlighted in red boxes. **(B)** An example of using HANDS to assign subgenome-specific base-identity at HSP positions. HANDS uses the read alignments from the sequencing alignment/mapping (SAM) file of the polyploid in conjunction with the bases in the diploid progenitor-relatives at HSP positions to assign variant bases to the polyploid subgenomes. In the example shown, the polyploid genome contains HSPs at positions 5 (A/C), 12 (C/T), 40 (C/G), 55 (A/C/T) and 92 (C/G). The reference genome is depicted in black and the RNA-seq reads aligned against the reference genome are shown as arrows below the reference genome. Bases in the reads matching the reference are shown in grey, while nucleotides at polymorphic sites are indicated by a capital letter. Read alignments for each gene are considered in turn from the SAM file and are processed in five steps: (1) creation of base patterns from aligned reads, (2) removal of potential sequencing errors and embedded base patterns, (3) assignment of base patterns to subgenomes, (4) assignment of bases to subgenomes using the previously assigned base patterns, and (5) assignment of bases to subgenomes using base patterns discarded in step 4. These steps are explained in greater depth in the main text and illustrated in the Note S1 in Additional file 1.

Bread wheat (*Triticum aestivum*), one of the world’s major crop plants, is an important example polyploid. The bread wheat genome is hexaploid, comprised of three distinct A, B and D subgenomes (AABBDD), and originated from two successive cross-hybridization events. First, ~0.5 million years ago, the tetraploid *Triticum turgidum* (AABB) arose from cross-hybridization between an A-genome donor diploid (closely related to extant *Triticum urartu*, AA) and a B-genome donor diploid (currently unknown but related to the wild goatgrass *Aegilops speltoides*, BB). Second, ~8,000 years ago, *T. aestivum* emerged from cross-hybridization between *T. turgidum* and another wild diploid goatgrass (*Aegilops tauschii*, DD) [7,8]. The A, B and D subgenomes of bread wheat are themselves derived from a common ancestor [9], and hence share extensive sequence relatedness (Additional file 1: Figure S1). This sequence relatedness makes it difficult to precisely assign individual gene fragments or transcripts to their subgenome of origin, and in turn makes determination of an accurate bread wheat reference genome sequence problematic. Nevertheless, despite their extensive sequence relatedness, the A, B and D genomes have diverged in sequence from their common ancestor, resulting in Homeolog-Specific Polymorphisms (HSPs). HSPs are the positions in a polyploid genome where the homeologous subgenomes have different bases (Figure 1A), and have often been used in transcriptomic and evolutionary studies, and to characterize homeolog-specific gene-expression [2,4,6]. However, these previous studies have involved only a limited number of genes, with genome-wide approaches being precluded by both genomic complexity and cost [10].

The recent advent of next-generation sequencing technology now makes it possible to survey multiple HSPs on a genome-wide basis [9], and to determine the homeologous subgenome-identity of individual bases at those HSP sites (Additional file 1: Figure S2). Thus, we have developed ‘HSP base Assignment using NGS data through Diploid Similarity’ (HANDS) as a method to characterize HSPs in polyploid genomes. In essence, HANDS involves comparative alignments of next-generation sequencing reads from polyploid and diploid progenitor-relatives onto a suitable reference sequence. Similarity of polyploid subgenomes with progenitor-relative diploids has recently been exploited to classify gene assemblies in bread wheat subgenomes [9] and sequencing reads in allopolyploid cotton [11], thus suggesting the viability of this approach for homeolog classification. In contrast, HANDS classifies individual HSP positions, and provides an added advantage in that it works even if only EST or mRNA datasets are available for polyploid genomes, thus allowing base characterization without the need of genomic assemblies.

Although HANDS was developed with bread wheat in mind, it is applicable to all allopolyploid genomes, and relies on sequence similarity between the polyploid subgenomes and the corresponding diploid progenitors. In the specific case of wheat HANDS uses the sequence similarity between the three A, B and D bread wheat subgenomes and the genomes of three extant diploid relatives of the original subgenome donors (Additional file 1: Figure S2). When applied to high-throughput RNA sequencing (RNA-seq) for bread wheat, HANDS correctly predicts subgenome-specific base-identity at over 90% of assayed HSPs. Thus HANDS allows rapid and accurate discovery of homeolog-specific base-identity at a genome-wide level.

## Results

### The HANDS framework

HANDS uses the sequencing alignment/mapping (SAM) file [12] of the polyploid genome (for example, bread wheat) obtained using sequence alignment tools such as Burrows-Wheeler Aligner [13] along with the variants lists (HSPs for the polyploid and single base substitutions (SBSs) for the progenitor-relative diploids) to assign base-identity to the polyploid subgenomes. The variant lists are tab-separated files containing sequence name, position, reference base and consensus base, and can be generated using tools such as SAMtools [12] (see Methods).

HANDS starts by optionally preprocessing the data to validate the HSPs and SBSs identified in the polyploid and progenitor-relative diploid genomes respectively. The preprocessing step is described in detail in Note S1 in Additional file 1. Once the preprocessing is done, HANDS considers the read alignments (from SAM file) for each gene simultaneously and proceeds in five steps (Figure 1B). In step 1, HANDS creates a base pattern for each read and counts the reads containing a particular base pattern. In step 2, base patterns that are a result of sequencing error or embedded in other base patterns are removed. In step 3, base patterns are assigned to one or more subgenomes based on their similarity with the corresponding diploid. In step 4, base patterns are prioritized based on their percentage identity with the diploids, and bases are assigned to each subgenome iteratively discarding base patterns that contradict with the already assigned bases. In step 5, the base patterns discarded in step 4 are re-used to characterize bases at unassigned positions. These steps are described in the next five subsections and are further explained in Note S1 in Additional file 1 using a detailed example.

#### Step 1: Creation of base patterns from aligned reads

In step 1, HANDS considers each read aligned against the current gene in turn and creates a base pattern for that read. A base pattern is regarded as the sequence of the pairs (position, nucleotide) for all HSPs found in the read. For paired-end data, alignments corresponding to both reads in the pair are considered together to generate a single base pattern for the pair. Read pairs, where a nucleotide discrepancy is found at a particular HSP position in the forward and reverse reads are ignored as this suggests that one of the two reads had a sequencing error (Additional file 1: Figure S3). Note that this scenario will only occur when reads in a pair have some overlap between them. While creating the base patterns, HANDS groups the same base patterns together keeping track of the number of reads supporting a particular base pattern. Once base patterns are obtained all further processing is done on these patterns and not on reads.

#### Step 2: Removal of potential sequencing errors and embedded base patterns

To remove the base patterns arising due to sequencing errors, HANDS calculates the base pairs (two consecutive HSPs) in each base pattern along with the number of reads supporting each pair. Base patterns containing a base pair that is present in less than 5% (a user-specified parameter) of the reads at these positions are ignored. HANDS further removes those base patterns that are embedded within another base pattern.

#### Step 3: Assignment of base patterns to individual genomes

Once a list of valid base patterns is obtained for the current gene, HANDS assigns the base patterns to individual genomes using the SBS data for the diploid progenitors. A base pattern is assigned to a genome if at least 50% (a user-specified parameter) of its bases at HSP positions match with the corresponding diploid genome. When calculating the percentage identity, positions with low coverage in the diploid (less than 3 (a user-specified parameter) reads) as well as positions where a unique nucleotide is not present (for example, heterozygous base substitution) are ignored.

#### Step 4: Assignment of bases to individual genomes

In step 4, HANDS assigns bases to a genome iteratively using the patterns assigned to the genome in step 3. For each HSP position, HANDS tabulates all nucleotides that are present in different base patterns assigned to an individual genome along with the base pattern(s) having the maximum percentage identity with the diploid genome containing that particular nucleotide. If only one nucleotide is present at a particular position, it is assigned at that position. Otherwise the nucleotide corresponding to the base pattern with the maximum percentage identity with the diploid progenitor is assigned at that position. If multiple nucleotides have a base pattern with the maximum percentage identity with the diploid then no assignment is made. Once all the positions have been checked, base patterns that contradict with the assigned positions are removed and the process is continued until no more positions can be assigned.

#### Step 5: Assignment of bases to individual genomes using discarded base patterns

HANDS finally uses the base patterns that were discarded in Step 4 from different genomes because they contradicted the assigned bases (see above) to correct the bases assigned to the genomes. The base patterns and subsequently bases are assigned to the genome using the same strategy as above but now the percentage identity is calculated using the previously assigned bases instead of diploid data. A nucleotide is only changed at a particular HSP position if the base pattern containing that nucleotide has a better percentage identity than the base pattern corresponding to the currently assigned nucleotide.

### Provision for distant genomes

HANDS also caters for the scenario where one diploid progenitor may be relatively distant from the corresponding genome in the polyploid. One such example is the B genome in wheat. The donor of the B genome is unknown but is hypothesized to be similar to *Aegilops speltoides*[7-9]. In this scenario, a base pattern may have very low or no identity with the diploids. HANDS uses the fact that the base pattern must come from one of the subgenomes of the polyploid and consequently assigns the base pattern that is not assigned to any genome (due to low identity with the diploids) in step 3 to the genome designated as the distant genome.

### Characterization of subgenome-specific base-identity in bread wheat

We used HANDS to characterize the HSPs in bread wheat (*T. aestivum* cv. Chinese Spring (CS)). To avoid the complexity due to the large genome size (16,000 Mb) and high repetitive content (>80%) of wheat [14,15], we sequenced the transcriptomes of *T. aestivum* (hexaploid), *T. urartu* (diploid A-genome progenitor-relative), *Ae. speltoides* (diploid B-genome progenitor-relative) and *Ae. tauschii* (diploid D-genome progenitor-relative) using Illumina paired-end sequencing technology (Additional file 1: Table S1, Methods), and aligned the resultant reads onto a wheat transcriptome reference sequence (Additional file 1: Table S2). This wheat transcriptome reference was constructed using Wheat UniGene Build 60 (http://www.ncbi.nlm.nih.gov/UniGene/) by concatenating the UniGenes such that they were separated by 200 ‘N’s (Additional file 1: Figure S4). The aligned reads were filtered to remove low quality alignments and variants (HSPs and SBSs for polyploid and diploid progenitor-relatives respectively) were called (Additional file 1: Table S2, Methods). HANDS was then used to characterize the HSPs in *T. aestivum* based on the similarity with the SBSs in the diploid progenitor-relatives. Out of a total of 342,266 HSP positions identified in *T. aestivum*, HANDS assigned bases at 325,517 positions (95.1%) to one or more subgenomes (Additional file 2: Table S3). Out of the remaining 16,749 positions where base assignments could not be made 16,454 (98.24%) positions were such that one or more diploids had low coverage or an ambiguous base (a heterozygous base substitution) was present at these positions and were consequently ignored.

### Assessment of HANDS base-identity assignment accuracy

To test the accuracy of this approach, we used *T. aestivum* nullisomic-tetrasomic (NT) lines. NT lines are a set of lines each missing a single chromosome (nullisomic) which is substituted by an additional copy of a homeologous chromosome (tetrasomic) [16], and provide an ideal framework to characterize wheat HSPs at the genome-wide level as described in Note S2 in Additional file 1. We focused our test on two of the seven chromosome groups of bread wheat by analyzing RNA-seq data from wheat lines nullisomic for individual group 1 (1A, 1B or 1D) and 5 (5A, 5B or 5D) homeologs using Illumina paired-end technology (Additional file 1: Table S1). As before, sequencing reads were aligned and filtered, and variants lists were generated (Additional file 1: Table S2, Methods). We then identified the A, B and D subgenome-specific bases corresponding to chromosomes 1 and 5 using NT lines (Methods, Note S2 in Additional file 1) and compared these homeologous assignments against those generated by HANDS (Table 1, Additional file 3: Table S4 and Additional file 4: Table S5). A total of 32,993 and 40,783 HSPs were identified on chromosomes 1 and 5, respectively. Positions where the diploid had low Illumina sequencing read coverage or an ambiguous base as well as positions where base assignments could not be made using NT lines or the subgenome was silenced (as identified by NT lines) were ignored and remaining positions were used for evaluating the accuracy of HANDS (Table 1). HANDS characterized bases to the subgenomes with high accuracy for both chromosomes (96.27%, 93.78% and 97.91% for A, B and D genome respectively on chromosome 1, and 96.58%, 93.88% and 97.81% for A, B and D genome respectively on chromosome 5) with only a very small fraction of incorrectly assigned positions (between 1.63% and 4.56%) and unassigned positions (between 0.46% and 1.66%).

**Table 1:** Performance evaluation of HANDS

	**Chromosome 1**			**Chromosome 5**		
	**A**	**B**	**D**	**A**	**B**	**D**
**HSP positions**	**32,993**	**32,993**	**32,993**	**40,783**	**40,783**	**40,783**
Positions ignored	4,669	5,540	4,205	6,230	7,284	4,420
Diploid coverage low	831	1,318	523	969	1,768	753
Diploid ambiguous	45	202	59	60	1,095	77
NT unassigned	2,627	2,627	2,627	2,866	2,866	2,866
Sub genome silenced	1,166	1,393	996	2,335	1,555	724
**Position considered**	**28,324**	**27,453**	**28,788**	**34,553**	**33,499**	**36,363**
Correct assignment	27,268 (96.27%)	25,746 (93.78%)	28,185 (97.91%)	33,371 (96.58%)	31,448 (93.88%)	35,565 (97.81%)
Incorrect assignment	882 (3.11%)	1,252 (4.56%)	470 (1.63%)	966 (2.80%)	1,518 (4.53%)	598 (1.64%)
No assignment	174 (0.61%)	455 (1.66%)	133 (0.46%)	216 (0.63%)	533 (1.59%)	200 (0.55%)

HSP bases were characterized for wheat chromosomes 1 and 5 using the *T. aestivum* nullisomic-tetrasomic lines and compared against the results obtained from HANDS. HANDS assigned the subgenome-specific base-identify with accuracy between 94% and 98%.

**Figure 2:**
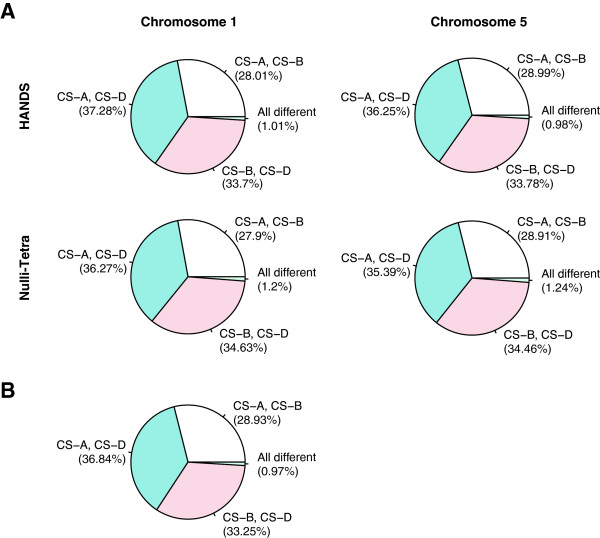
**Percentages of shared bases between different *T. aestivum *subgenomes. (A)** Percentages of shared bases between different *T. aestivum* subgenomes at HSP positions calculated using the base-identities assigned through NT lines and predicted by HANDS on chromosomes 1 and 5. **(B)** Percentages of shared bases calculated using the base-identities predicted by HANDS for the complete *in silico* reference genome. In all cases, the percentages were calculated by considering HSP positions where base-identity was assigned to all three subgenomes (25,259 on chromosome 1; 30,433 on chromosome 5; and 249,975 on complete *in silico* reference).

**Figure 3:**
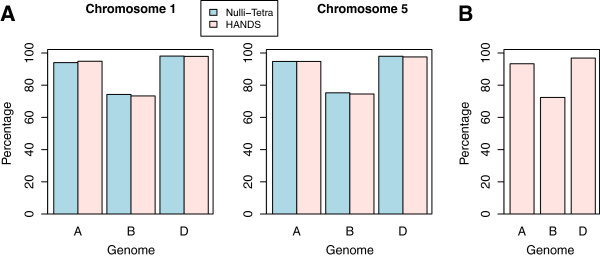
**Percentages of shared bases between *T. aestivum *subgenomes and corresponding diploid progenitor-relatives. (A)** Percentages of shared bases between *T. aestivum* subgenomes and corresponding diploid progenitor-relatives at HSP positions calculated using the base-identities assigned through NT lines and predicted by HANDS on chromosomes 1 and 5. **(B)** Percentages of shared bases calculated using the base-identities predicted by HANDS for the complete *in silico* reference genome. In all cases, the percentages were calculated by considering HSP positions where base-identity was assigned to all three subgenomes (25,259 on chromosome 1; 30,433 on chromosome 5; and 249,975 on complete *in silico* reference).

We further compared the percentages of bases shared between different subgenomes of wheat, and between wheat subgenomes and corresponding diploid progenitors using the base-identities assigned through NT lines and predicted using HANDS for chromosomes 1 and 5 to see if the patterns were consistent. The results are shown in Figures 2A and 3A. In both cases, the differences in the percentages were not significant (Fisher’s exact test, P-values = 1).

## Discussion

We have presented HANDS, a method for rapid and accurate characterization of HSPs in polyploid genomes. HANDS uses next-generation sequencing data from the polyploid and the diploid progenitors to characterize bases in the polyploid genome at HSP positions without requiring to sequence a large number of genomes. This not only reduces the cost associated with genome sequencing but also eliminates the need of generating NT lines for each chromosome of the polyploid genome. The current implementation allows characterization of base-identities in any allopolyploid containing up to three subgenomes and is therefore applicable to a number of important polyploid genomes such as *Brassica napus* (rapeseed) [17], *Gossypium hirsutum* (cotton) [18] and *Avena sativa* (oat) [19]. Although, the results are presented in this paper using SAM file generated by BWA and variant lists generated by SAMtools, it is important to note that HANDS is independent of the sequence mapping and variant calling software, and can characterize HSPs using output from any standard mapping/variant calling tool.

When tested on chromosomes 1 and 5 of bread wheat, HANDS characterized bases to the subgenomes with high accuracy for both chromosomes. However, for both chromosomes 1 and 5, the base assignment accuracy was lowest for the B subgenome (Table 1). This is likely due to the fact that *Ae. speltoides*, used as the B genome diploid progenitor-relative for base assignments, is actually more distantly related to the *T. aestivum* B subgenome than is the case for the A and D genome progenitor relatives [7-9]. Nevertheless, HANDS still assigns bases with great accuracy (~94%) to the B subgenome, because it caters for the scenario where one progenitor may be relatively distant from the corresponding polyploid subgenome when assigning bases (see above).

Given this defined level of accuracy for group 1 and group 5 chromosomes, it is likely that HANDS works with >90% accuracy throughout the entire bread wheat genome. To further support this proposition, we calculated the percentage of shared bases between different subgenomes of wheat, and between wheat subgenomes and corresponding diploid progenitors across the whole *in silico* reference using the base-identities predicted by HANDS, and compared the results to those obtained for chromosomes 1 and 5 (Figures 2B and 3B). No significant difference was found between the full and partial datasets (Fisher’s exact test, P-values ≈ 1).

We next sought to calculate the percentage of HSPs that were tri-homeoallelic (i.e. positions containing three different alleles) in our datasets. It has been recently reported that the percentage of tri-homeoallelic HSPs present in bread wheat is surprising low although it contains three homeologous subgenomes [20]. We found that the percentages of tri-homeoallelic HSPs in partial (chromosomes 1 and 5) as well as full datasets were comparable to those reported earlier (Additional file 1: Figure S5). This further supports the proposition that HANDS works with high accuracy across the whole *in silico* reference. Moreover, it is possible that HANDS classification of individual HSPs accounts for the greater accuracy of this method (exploiting the similarity of the polyploid subgenomes with the diploid progenitor relatives) versus the previous gene-assembly based method, which respectively gave accuracies of ~72% for the A, ~85% for the D and ~60% for the B genomes [9].

## Conclusion

In summary, HANDS allows accurate genome-wide discovery of subgenome-specific base-identity in polyploids. In the specific case of wheat, this advance will enhance our understanding of the complex genome architecture of this important crop plant. In particular, it provides the ability to relate, on a genome-wide basis, specific homeoallelic variants to particular agronomic traits, thus increasing the precision of crop-breeding solutions to address the challenge of global food security.

## Methods

### Plant material

Three spikelets of *T. aestivum* CS, *T. urartu*, *Ae. speltoides* and *Ae. tauschii* and chromosomes 1 and 5 Nullisomic-Tetrasomic (NT) lines were placed on Whatman filter papers soaked with water in Petri-dishes. The spikelets were stratified at 4°C for 2 days in the dark before extracting the seeds from the spikelets. The seeds were then transferred back to 4°C for a further 2 days after which they were allowed to grow at room temperature. Three replicates for each plant were then grown hydroponically in a controlled environment room (16/8-h light/dark cycle at 21°C, irradiance 120 μmol photons m^-2^ s^-1^). Root and shoot tissue samples were collected when the fifth leaf appeared. At this point, the fourth leaf was taken as the shoot sample.

### RNA extraction and sequencing

RNA was extracted from the shoot and root samples using Trizol (Invitrogen) using the standard protocol. The Illumina Genome Analyzer II (GAII) platform was used for transcriptomic sequencing, according to the manufacturer’s instructions, at the Wellcome Trust Centre for Human Genetics, Oxford, UK. Four lanes of 36, 51 and/or 71 bp paired-end data were generated for diploids and NT lines whereas two lanes of 100 bp paired-end data were generated for CS for both root and shoot samples of a single plant. Between 244.6 and 694.9 million reads were produced for each line (Additional file 1: Table S1).

### Sequence alignment, filtering and visualization

Sequencing reads for *T. aestivum* CS*, T. urartu, Ae. speltoides, Ae. tauschii*, and chromosome 1 and 5 NT lines were mapped to the *in silico* reference genome created using Wheat UniGene Build 60 (http://www.ncbi.nlm.nih.gov/UniGene/, Additional file 1: Figure S4) using Burrows-Wheeler Aligner (BWA) [13] using default parameters. Reads with low mapping quality (phred score ≤ 20), reads for which the read pair did not map onto the reference genome, and reads that did not map uniquely onto the reference genome were removed from subsequent analyses using custom scripts written in C++. The alignments were visualized using Integrated Genome Viewer (IGV) [21].

### Variant calling and filtering

The filtered alignments were used to generate HSP and SBS lists for the polyploid and diploids respectively using SAMtools [12] (Additional file 1: Table S2). First, pileups were generated using ‘mpileup’ command with probabilistic realignment for the computation of base alignment quality (BAQ) disabled and maximum coverage threshold set to 50,000. These pileups were then used to call variants using bcftools (available as part of SAMtools) using default parameters. The HSP and SBS lists were filtered to remove potential false positives including those due to errors arising during DNA sequencing itself. Variants with low quality (phred score ≤ 20) as well as those with read coverage of less than 3 reads per site were removed. For diploids, ambiguous base calls were also ignored.

### Identification of chromosomes 1 and 5 UniGenes

To identify the UniGenes located on chromosomes 1 and 5, we first characterized HSP bases for all 56,954 UniGenes present in the Wheat UniGene build 60 separately for chromosomes 1 and 5 using NT lines (Note S2 in Additional file 1). For each chromosome, we then extracted those UniGenes for which base assignments were made for at least 60% of the HSPs present in the UniGene. This process identified 2,773 and 3,218 UniGenes on chromosomes 1 and 5 respectively. HSPs present on these UniGenes were used for the evaluation of HANDS’s performance.

### Availability of supporting data

The Illumina sequencing data files have been submitted to the NCBI Sequence Read Archive (SRA) (http://www.ncbi.nlm.nih.gov/sra) under accession number SRA097144. HANDS is implemented in Java and is available online (http://lums.edu.pk/sse/biology/aziz-mithani-hands/).

## Competing interests

The authors declare no competing interests.

## Authors’ contributions

AM and NPH conceived HANDS with EJB and CJ providing further initial input. AM, EJB, and CB grew plants, extracted RNA and commissioned sequencing. AM developed and implemented HANDS. LL provided addition bioinformatics support. EJB, CJ, and NPH provided additional input. AM and NPH wrote the manuscript. All others read and approved the final manuscript.

## Supplementary Material

Additional file 1: Figure S1 Percentage of Polymorphic bases in *T. aestivum*. **Figure S2.** Identification of HSPs using next-generation sequencing data. **Figure S3.** Nucleotide discrepancy between paired-reads. **Figure S4.** Creation of *in silico* reference using the UniGene set. **Figure S5.** Percentage of tri-homeoallelic HSPs. **Table S1.** Genome sequencing summary for *T. aestiuvm* CS, *T. urartu*, *Ae. speltoides*, *Ae. tauschii*, and chromosome 1 and 5 nullisomic-tetrasomic lines. **Table S2.** Alignment mapping summary for *T. aestivum* CS, *T. urartu*, *Ae. speltoides*, *Ae. tauschii*, and chromosome 1 and 5 nullisomic-tetrasomic lines. **Note S1.** Base characterization using HANDS. **Note S2.** Base characterization using nullisomic-tetrasomic lines. Click here for file

Additional file 2: Table S3 HSP characterization across the whole *in silico* reference using HANDS. Click here for file

Additional file 3: Table S4 HSP characterization using HANDS and NT lines for chromosome 1. Click here for file

Additional file 4: Table S5 HSP characterization using HANDS and NT lines for chromosome 5. Click here for file
