# Supplementary material for: HANDS: a tool for genome-wide discovery of subgenome-specific base-identity in polyploids
Source: BMC Genomics. 2013 Sep 24;14:653. doi: 10.1186/1471-2164-14-653 (PMC3849207; doi:10.1186/1471-2164-14-653)
Supplement: Additional file 1: Figure S1 — Percentage of Polymorphic bases in T. aestivum. Figure S2. Identification of HSPs using next-generation sequencing data. Figure S3. Nucleotide discrepancy between paired-reads. Figure S4. Creation of in silico reference using the UniGene set. Figure S5. Percentage of tri-homeoallelic HSPs. Table S1. Genome sequencing summary for T. aestiuvm CS, T. urartu, Ae. speltoides, Ae. tauschii, and chromosome 1 and 5 nullisomic-tetrasomic lines. Table S2. Alignment mapping summary for T. aestivum CS, T. urartu, Ae. speltoides, Ae. tauschii, and chromosome 1 and 5 nullisomic-tetrasomic lines. Note S1. Base characterization using HANDS. Note S2. Base characterization using nullisomic-tetrasomic lines. [file 1471-2164-14-653-S1.doc]

**HANDS: A tool for genome-wide discovery of subgenome-specific base-identity in polyploids**

Aziz Mithani et al.

| **Item** | **Description** |
| --- | --- |
| Figure S1 | Percentage of Polymorphic bases in *T. aestivum* |
| Figure S2 | Identification of HSPs using next-generation sequencing data |
| Figure S3 | Nucleotide discrepancy between paired-reads |
| Figure S4 | Creation of *in silico* reference using the unigene set |
| Figure S5 | Percentage of tri-homeoallelic HSPs |
| Table S1 | Genome sequencing summary for *T. aestiuvm* CS, *T. urartu*, *Ae. speltoides*, *Ae. tauschii*, and chromosome 1 and 5 nullisomic-tetrasomic lines |
| Table S2 | Alignment mapping summary for *T. aestivum* CS, *T. urartu*, *Ae. speltoides*, *Ae. tauschii*, and chromosome 1 and 5 nullisomic-tetrasomic lines |
| Note S1 | Base characterization using HANDS |
| Note S2 | Base characterization using nullisomic-tetrasomic lines |

**Note:**

Supplemental Tables S3, S4 and S5 are provided as Excel files.

**Figure S1. Percentage of polymorphic bases in *T. aestivum*.** Polymorphic bases correspond to positions where at least one of the three subgenomes has a different base than the remaining subgenomes. The percentages were calculated by considering positions in the *in silico* reference (Supplemental Fig. S3) where there was a coverage of three or more RNA-seq reads.

**
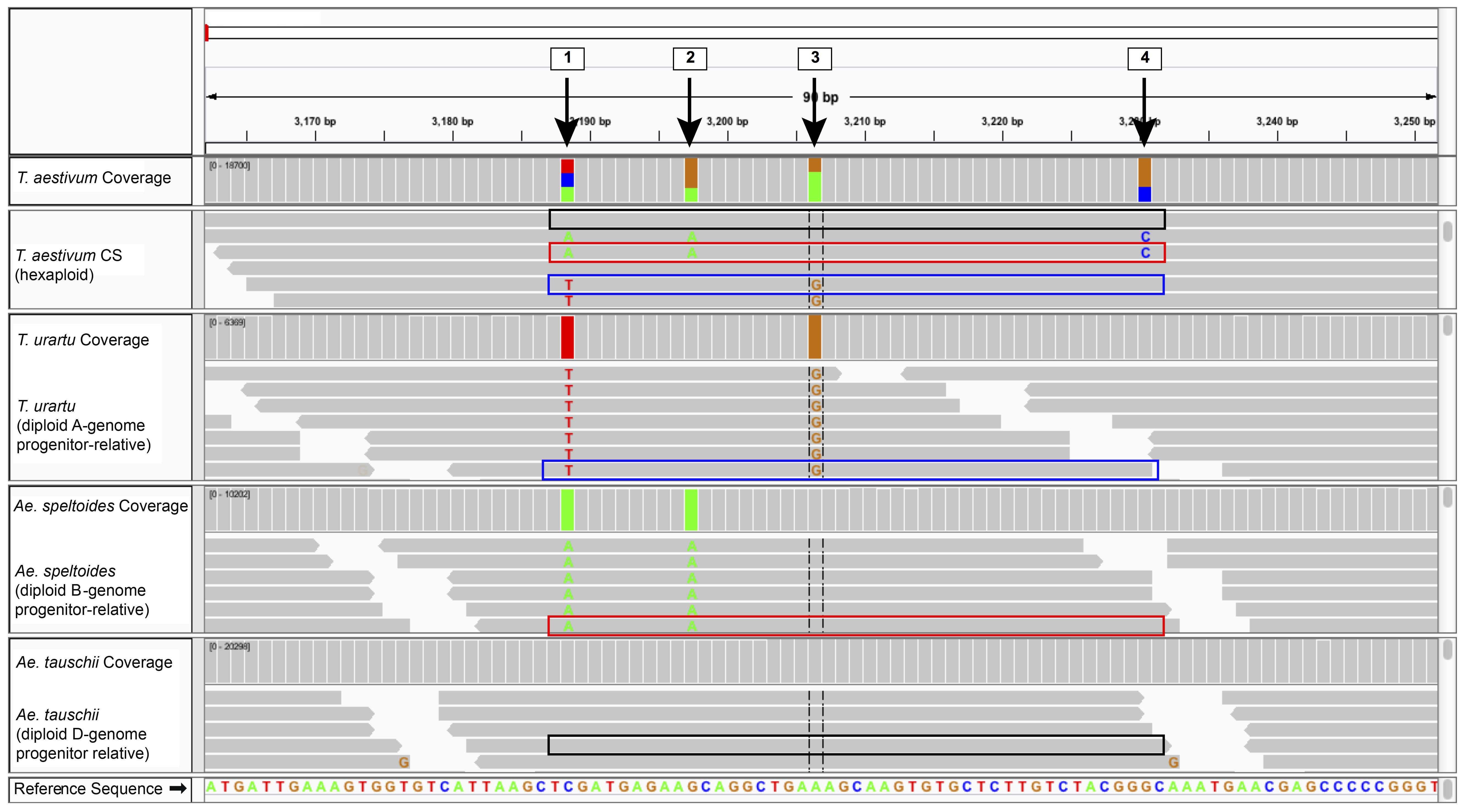
**

**Figure S2. Identification of HSPs using next-generation sequencing data.** Figure shows Integrated Genome Viewer (IGV) screenshots of RNAseq data of *T. aestivum* cv. Chinese Spring (CS), *T. urartu* (A-genome progenitor-relative), *Ae. speltoides* (B-genome progenitor-relative) and *Ae. tauschii* (D-genome progenitor-relative) generated using Illumina paired-end sequencing technology. Bases that match the reference sequence are grey and base substitutions (versus the reference sequence) are shown in other colors. (1) *T. aestivum* CS sequencing read aligned to reference sequence reveal the bases A, C and T at the HSP position. Diploid data suggests that the reads containing the base T are from the A-subgenome, reads containing the base Aare from the and B-subgenome and the reads containing the base C are from the D-subgenome; (2) *T. aestivum* CS sequencing read aligned to reference sequence reveal the bases A and G at the HSP position. Diploid data suggests that the reads containing the base G are from the A- and D-subgenomes, whereas the reads containing the base A are from the B-subgenome; (3) *T. aestivum* CS sequencing read aligned to reference sequence reveal the bases A and G at the HSP position. Diploid data suggests that the reads containing the base A are from the B- and D-subgenomes and the reads containing the base G are from the A-subgenome; (4) An example of a polyploid-specific HSP which has no corresponding base substitutions in the diploids. Bases can be assigned to individual subgenomes of the polyploid genome based on the similarity with the diploid genomes shown by different colored boxes (blue for A-subgenome, red for B-subgenome and black for D-subgenome). Although position 4 has no corresponding base substitutions in the diploids, using all HSPs together it is possible to assign the bases to individual subgenomes based on similarity with the diploids. Consequently, the base C is assigned to the B-subgenome (red box) and the base G is assigned to the other two subgenomes.

**
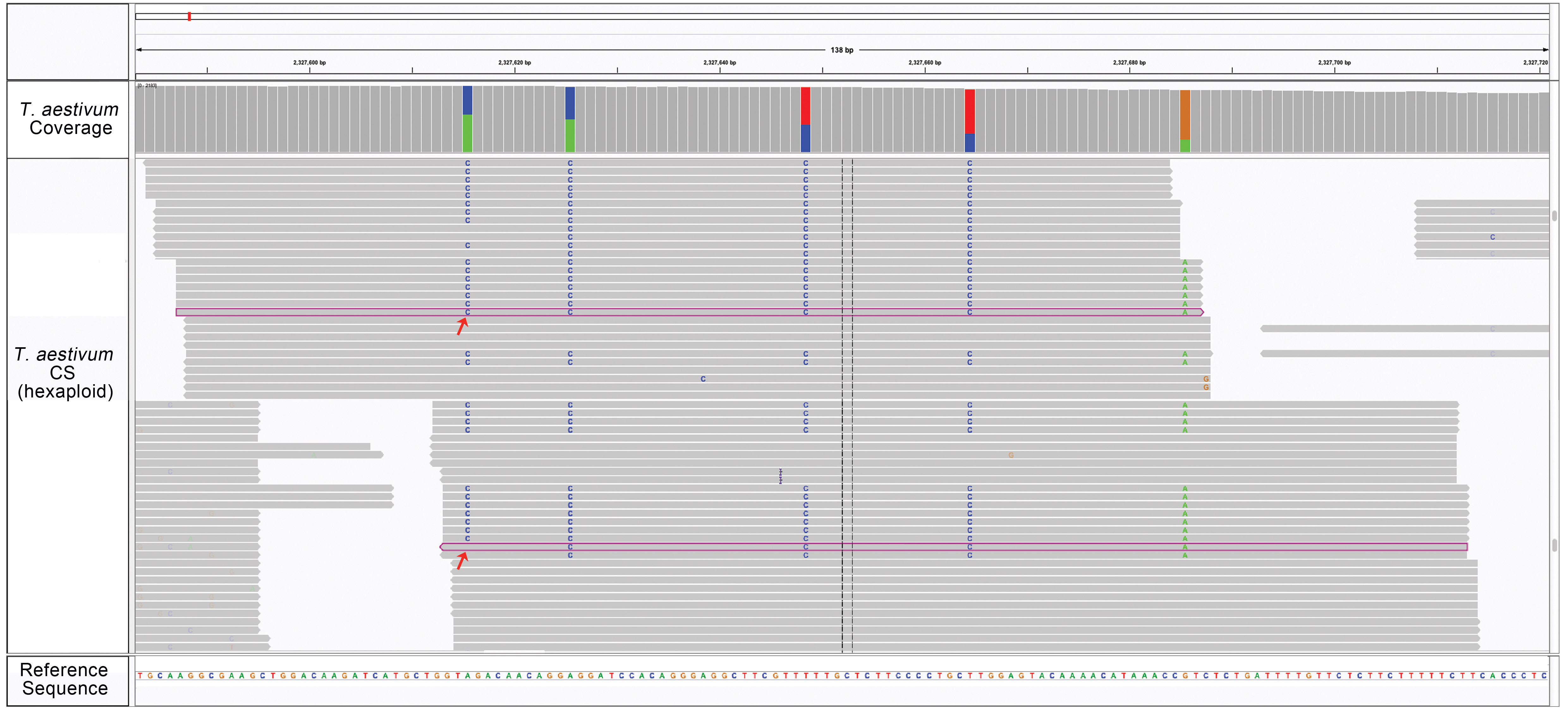
**

**Figure S3. Nucleotide discrepancy between paired-reads.** Figure shows Integrated Genome Viewer (IGV) screenshots of RNAseq data of *T. aestivum* cv. Chinese Spring (CS) generated using Illumina paired-end sequencing technology. Bases that match the reference sequence are grey and base substitutions (versus the reference sequence) are shown in other colors. The read-pair highlighted in purple has a discrepancy at the position marked with red arrows. The forward read contains a C at this position whereas the reverse read contains an A (same as the reference sequence). This is due to a sequencing error in one of the two reads. Read pairs with sequencing errors are ignored when creating base patterns.

**Figure S4. Creation of an *in silico* *T. aestivum* reference sequence.** A diagrammatic representation of the *T. aestivum* reference sequence used in our analyses. The reference was created by concatenating 56,954 *T. aestivum* UniGene sequences (*T. aestivum* UniGene Build 60) such that two consecutive UniGenes were separated by a gap of 200 N’s. The reference was a total of 61,117,745 bases in length out of which 11,391,000 bases were ‘N’s used as separators.

**Figure S5. Percentage of tri-homeoallelic HSPs.** Tri-homoeoallelic HSPs correspond to positions containing three different alleles. The reported percentages were obtained from Winfield et al (2012)[[1]](#footnote-2). For chromosomes 1 and 5, and the complete dataset, the percentages were calculated by considering HSP positions where base-identity was assigned to all three subgenomes (25,259 on chromosome 1; 30,433 on chromosome 5; and 249,975 on complete *in silico* reference).

**Table S1**

| **Line** | **Illumina paired-end read length (bp)** | **Total number of reads** | **Total number of bases sequenced** |
| --- | --- | --- | --- |
| *T. aestivum* CS | 51 | 479,164,998 | 24,437,414,898 |
| *T. urartu* | 51 | 298,503,992 | 15,223,703,592 |
| *Ae. speltoides* | 51 | 456,908,922 | 23,302,355,022 |
| *Ae. tauschii* | 51 | 244,632,212 | 12,476,242,812 |
| N1AT1B | 36 | 16,272,204 | 19,635,349,522 |
|  | 51 | 292,767,814 |  |
|  | 76 | 54,189,364 |  |
| N1AT1D | 51 | 435,406,078 | 22,205,709,978 |
| N1BT1A | 51 | 271560108 | 27,141,867,316 |
|  | 76 | 174,898,708 |  |
| N1BT1D | 36 | 15,857,786 | 19,551,553,458 |
|  | 51 | 372,170,062 |  |
| N1DT1A | 51 | 438,594,132 | 22,368,300,732 |
| N1DT1B | 51 | 319,917,776 | 28,471,256,912 |
|  | 76 | 159,940,136 |  |
| N5AT5B | 51 | 479,261,030 | 24,442,312,530 |
| N5AT5D | 51 | 443,198,748 | 22,603,136,148 |
| N5BT5A | 51 | 454,824,624 | 23,196,055,824 |
| N5BT5D | 51 | 492,792,280 | 25,132,406,280 |
| N5DT5A | 51 | 508,414,782 | 25,929,153,882 |
| N5DT5B | 51 | 598,385,686 | 30,517,669,986 |

Genome sequencing summary for *T. aestiuvm* CS, *T. urartu*, *Ae. speltoides*, *Ae. tauschii*, and chromosome 1 and 5 nullisomic-tetrasomic lines used in this study. RNA-seq data was generated for these lines using Illumina paired-end sequencing technology at the Wellcome Trust Centre for Human Genetics, Oxford, UK. Between 244.6 and 598.5 million reads were generated for each line resulting in between 12.4 and 30.5 billion bases being sequenced.

**Table S2**

| **Line** | **Total number of reads** |  | **Mapped data** | |  | **Filtered data** | |  | **Base substitutions** | |
| --- | --- | --- | --- | --- | --- | --- | --- | --- | --- | --- |
|  |  |  | **Number of reads** | **%** |  | **Number of reads** | **%** |  | **Reported** | **Accepted** |
| *T. aestivum CS* | 694,923,198 |  | 552,455,200 | 79.50 |  | 143,200,274 | 20.61 |  | 389,955 | 367,948 |
| *T. urartu* | 298,503,992 |  | 205,352,316 | 68.79 |  | 98,773,842 | 33.09 |  | 363,203 | 329,089 |
| *Ae. speltoides* | 469,436,158 |  | 387,437,226 | 82.53 |  | 96,074,712 | 20.47 |  | 468,048 | 381,336 |
| *Ae. tauschii* | 623,550,760 |  | 459,953,330 | 73.76 |  | 249,257,228 | 39.97 |  | 374,015 | 338,401 |
| N1AT1B | 363,229,382 |  | 256,390,430 | 70.59 |  | 148,238,814 | 40.81 |  | 494,049 | 460,598 |
| N1AT1D | 435,406,078 |  | 278,574,338 | 63.98 |  | 174,209,408 | 40.01 |  | 514,777 | 477,856 |
| N1BT1A | 446,458,816 |  | 312,691,682 | 70.04 |  | 191,222,970 | 42.83 |  | 516,649 | 487,200 |
| N1BT1D | 388,027,848 |  | 277,139,620 | 71.42 |  | 162,021,278 | 41.76 |  | 471,409 | 439,710 |
| N1DT1A | 438,594,132 |  | 327,945,010 | 74.77 |  | 182,181,736 | 41.54 |  | 528,538 | 492,024 |
| N1DT1B | 479,857,912 |  | 319,438,022 | 66.57 |  | 190,196,508 | 39.64 |  | 531,802 | 498,309 |
| N5AT5B | 479,261,030 |  | 364,945,100 | 76.15 |  | 154,398,178 | 32.22 |  | 523,421 | 490,207 |
| N5AT5D | 443,198,748 |  | 341,024,042 | 76.95 |  | 149,763,192 | 33.79 |  | 527,623 | 493,969 |
| N5BT5A | 454,824,624 |  | 345,728,126 | 76.01 |  | 155,492,412 | 34.19 |  | 520,702 | 487,430 |
| N5BT5D | 492,792,280 |  | 376,153,392 | 76.33 |  | 162,865,970 | 33.05 |  | 530,456 | 498,520 |
| N5DT5A | 508,414,782 |  | 393,180,466 | 77.33 |  | 178,119,122 | 35.03 |  | 524,574 | 493,168 |
| N5DT5B | 598,385,686 |  | 464,132,788 | 77.56 |  | 213,181,348 | 35.63 |  | 489,962 | 465,829 |

Alignment mapping summary for *T. aestivum* CS, *T. urartu*, *Ae. speltoides*, *Ae. tauschii*, and chromosome 1 and 5 nullisomic-tetrasomic lines used in this study. RNA-seq reads were mapped to the *in silico* reference sequence using BWA**[[2]](#footnote-3)**. Between 63% and 83% reads mapped against the reference sequence. The filtering process removed reads that had Phred-scaled mapping quality of 20 or less, reads for which the mate (the other read of a pair) did not map onto the reference genome, and reads that did not map uniquely onto the reference genome. As a result of the filtering process, between 20% and 43% reads were left in each line. Base substitutions were called using the filtered reads using SAMtools**[[3]](#footnote-4)**. Variants with coverage of greater than or equal to 3 reads or with Phred-scaled quality more than 20 were accepted. For diploids, ambiguous base calls were rejected. For polyploids (*T. aestivum* and NT lines), base substitutions include positions where all three subgenomes had the same base, which was different from the base in the reference sequence, in addition to HSPs.

# Note S1

# Base characterization using HANDS

## Data preprocessing

HANDS starts by optionally preprocessing the data to validate the HSPs and Single Base Substitutions (SBSs) in the polyploid and progenitor diploid genomes respectively. To do this, the HSPs and SBSs are first tabulated together, and then the base distributions obtained from SAM/BAM files are used to check if HSPs and SBSs are called correctly at the tabulated positions. HSPs may be missed by SAMtools due to low homeolog expression in one or more subgenomes. Similarly, diploid SBS lists are validated to check if the bases have been correctly called as base substitutions. This is done as follows. For the polyploid at least 5% of reads present at an HSP position must support an HSP base for it to be considered as present at that position. The threshold was selected to ensure that the false-positives arising due to misalignment of the sequencing reads are not regarded as valid HSPs. For the diploids at least 30% of the reads must support a base for it to be considered as a SBS. A considerably higher read percentage was required for the diploids the issue of low homeolog expression could not arise in the diploids.

After preprocessing, HANDS considers all read alignments (from the SAM file) for each gene simultaneously and proceeds in five steps (**Fig. 1**). These are described in in the main text and further explained using a detailed example below.

**Step 1: Creation of base patterns from aligned reads**

Consider the read alignments in the region between positions 680,037 and 681,191 corresponding to UniGene Ta.1023 shown below.


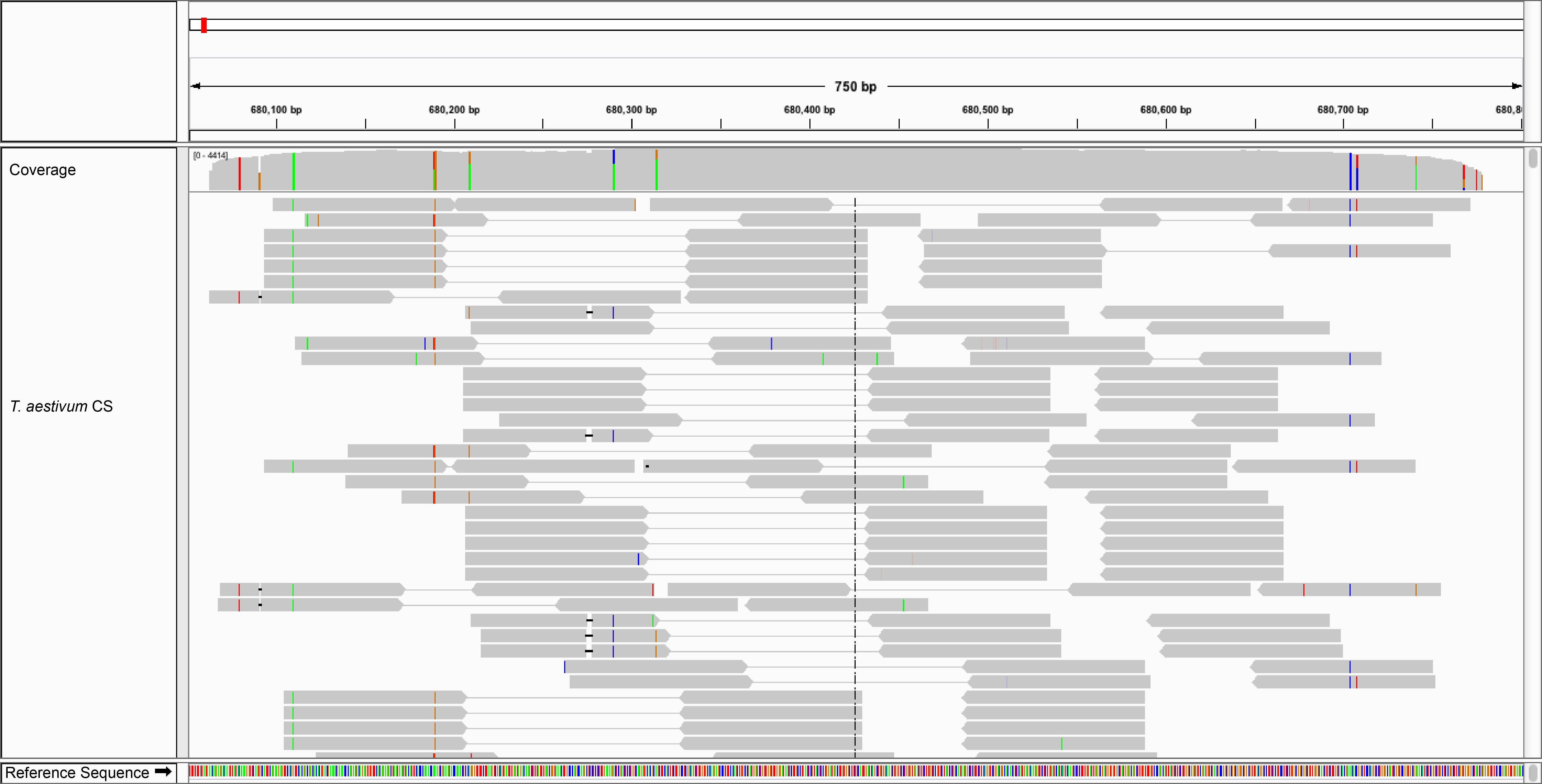


Bases that match the reference sequence are grey whereas the base substitutions are shown in other colors. As can be seen from the figure, the alignments correspond to paired-end data and, therefore, the base patterns are created from both reads in a pair in this case. Consider a part of the above alignment shown below.


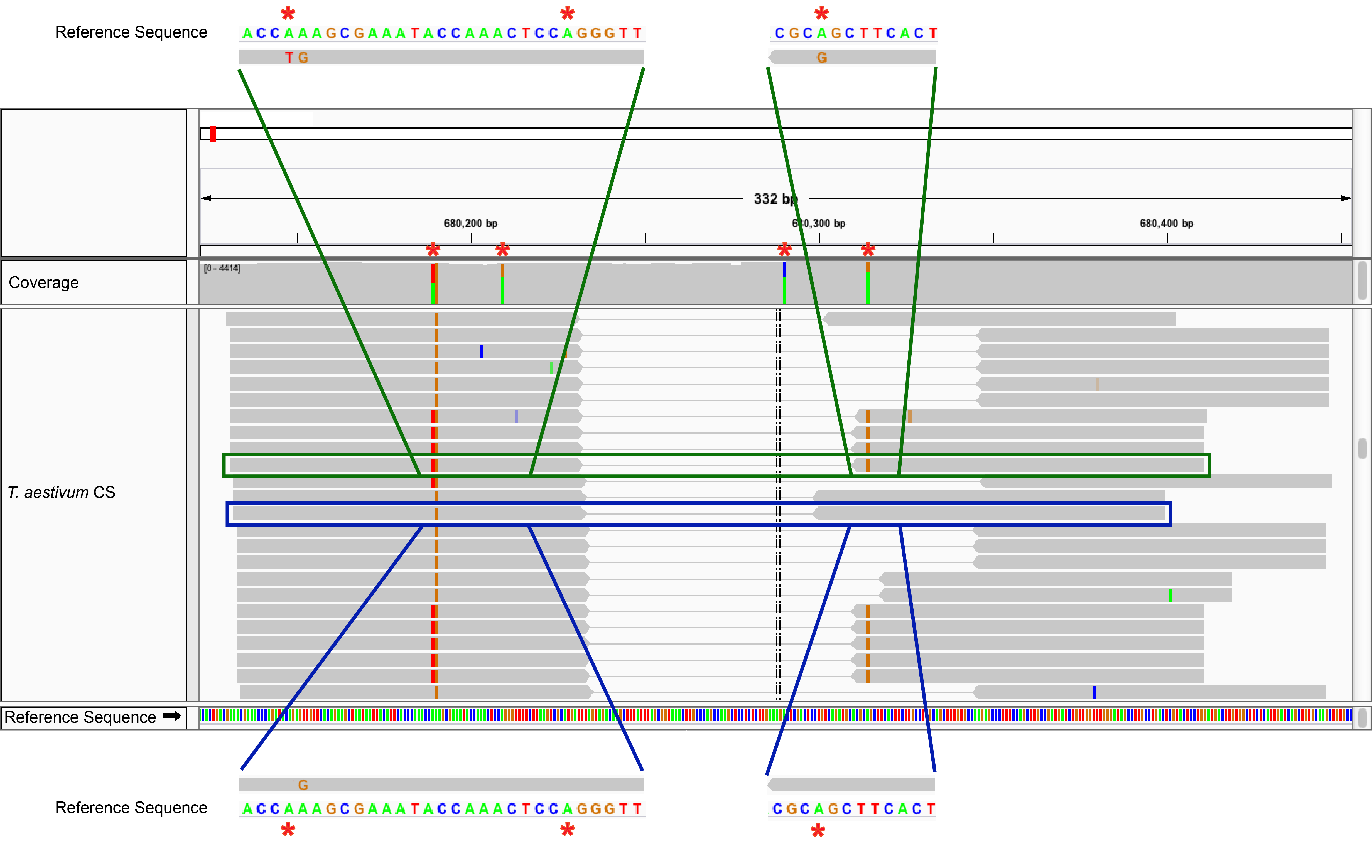


The HSPs are present at positions 680189, 680209, 680290 and 680314 marked with *. Note that there is the base G at position 680190 (compared to the base A in the reference) but it is not considered when creating the base pattern, as it is a base substitution with respect to the reference genome and not an HSP. This might be due either to varietal difference or to error in the UniGene set. The read-pairs highlighted with green and blue boxes cover three out of these four HSPs (680189, 680209 and 680314). The base pattern corresponding to the read-pair highlighted in green is given as follows.

{(680189,T), (680209,A), (680314,G)}

Similarly, the base pattern corresponding to the read-pair highlighted in blue is given as follows.

{(680189,A), (680209,A), (680314,A)}

Base patterns were created and tabulated in a similar fashion from remaining read-pairs mapped in the region between positions 680,037 and 681,191. This resulted in a total of 45 base patterns, which are listed below.

| ***T. aestivum* Base Patterns** | **No. of Reads** |
| --- | --- |
| {(680189,A)} | 40 |
| {(680189,T), (680209,A)} | 394 |
| {(680189,A), (680209,A)} | 401 |
| {(680189,A), (680209,A), (680290,A)} | 3 |
| {(680209,A), (680290,C)} | 125 |
| {(680290,A)} | 120 |
| {(680189,T), (680209,A), (680290,C)} | 4 |
| {(680290,C), (680314,G)} | 472 |
| {(680290,A), (680314,A)} | 1046 |
| {(680189,A), (680290,A), (680314,A)} | 275 |
| {(680189,T), (680209,A), (680290,C), (680314,G)} | 29 |
| {(680189,A), (680209,A), (680290,A), (680314,A)} | 11 |
| {(680189,T), (680290,C), (680314,G)} | 9 |
| {(680209,A), (680290,C), (680314,G)} | 1 |
| {(680189,T), (680209,G), (680290,C), (680314,G)} | 11 |
| {(680189,A), (680314,A)} | 208 |
| {(680314,G)} | 326 |
| {(680314,A)} | 317 |
| {(680189,T), (680314,G)} | 30 |
| {(680189,A), (680209,A), (680314,A)} | 70 |
| {(680189,T), (680209,A), (680314,G)} | 44 |
| {(680189,T), (680209,G), (680314,G)} | 24 |
| {(680189,T), (680209,G), (680314,A)} | 24 |
| {(680209,A), (680290,A), (680314,A)} | 2 |
| {(680189,T)} | 9 |
| {(680189,T), (680209,G)} | 313 |
| {(680209,A), (680290,A)} | 187 |
| {(680209,G), (680290,C)} | 247 |
| {(680209,A)} | 1 |
| {(680290,C)} | 67 |
| {(680209,G), (680290,A)} | 2 |
| {(680708,C)} | 306 |
| {(680708,T)} | 160 |
| {(680708,T), (680741,A)} | 217 |
| {(680708,C), (680741,G)} | 192 |
| {(680708,C), (680741,A)} | 184 |
| {(680708,C), (680741,A), (680768,G)} | 28 |
| {(680708,T), (680741,A), (680768,C)} | 1 |
| {(680708,C), (680741,G), (680768,G)} | 7 |
| {(680708,T), (680741,A), (680768,T)} | 25 |
| {(680708,C), (680741,G), (680768,C)} | 5 |
| {(680708,C), (680741,A), (680768,C)} | 6 |
| {(680708,C), (680741,G), (680768,G), (680773,C)} | 3 |
| {(680708,C), (680741,A), (680768,G), (680773,G)} | 14 |
| {(680708,T), (680741,A), (680768,T), (680773,C)} | 64 |

**Step 2: Removal of potential sequencing errors and embedded base patterns**

The following 5 base patterns from the list of base patterns obtained from the read-pairs were removed because one or more base pairs in these patterns were represented by less than 5% of reads.

- {(680209,G), (680290,A)}
- {(680708,T), (680741,A), (680768,C)}
- {(680708,C), (680741,G), (680768,C)}
- {(680708,C), (680741,A), (680768,C)}
- {(680708,C), (680741,G), (680768,G), (680773,C)}

For example, there were only 2 read pairs supporting the base pattern {(680209,G), (680290,A)}. The total number of read pairs covering positions 680209 and 680290 (shown in red in the above table) is 622. Since only 0.3% read pairs supported the base pattern, it was removed from the list.

The remaining 40 patterns were further checked to remove those patterns that were embedded in other patterns. For example, consider the base pattern {(680189,T), (680209,A), (680290,C), (680314,G)} corresponding to the read-pair highlighted in green in the following figure.


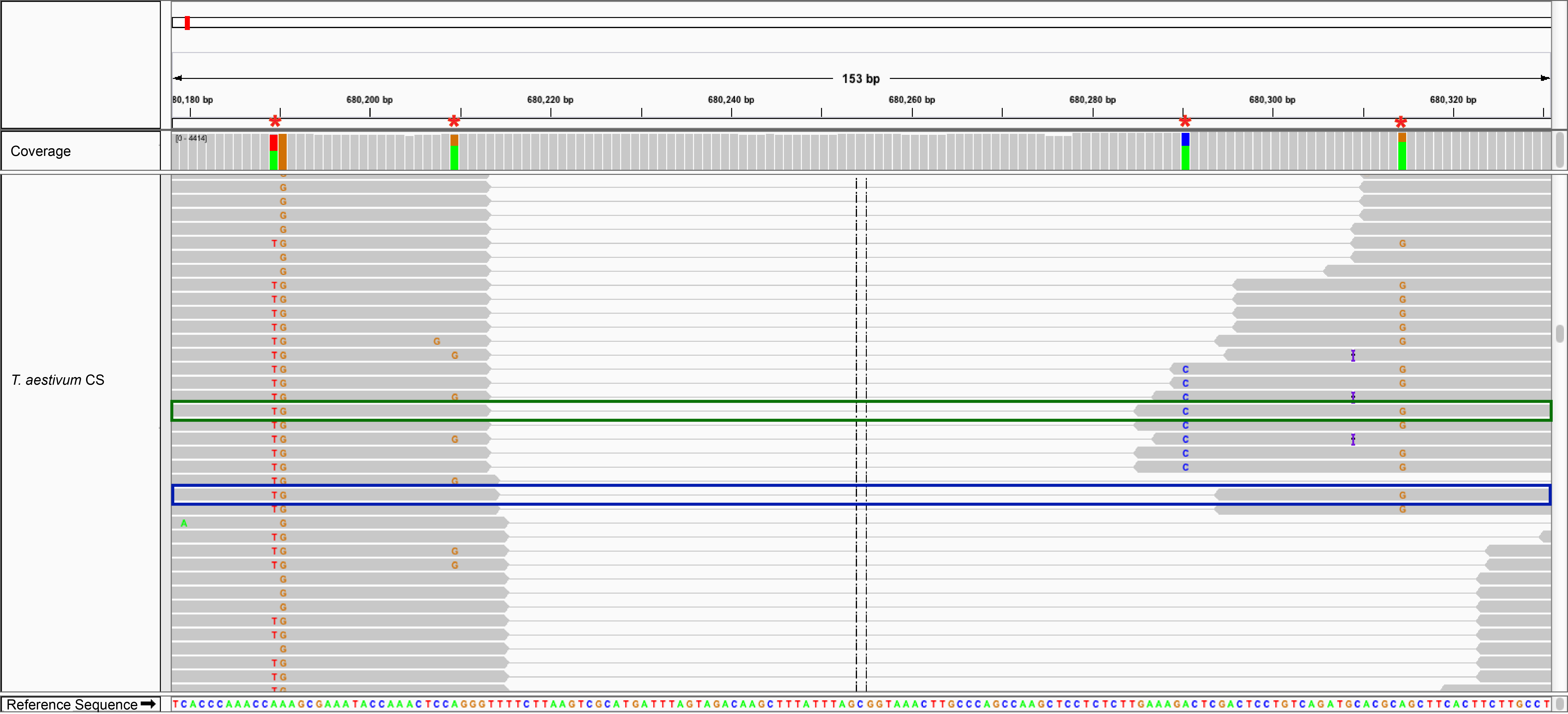


The base pattern {(680189,T), (680209,A), (680314,G)} corresponding to the read-pair marked in blue in the above figure is embedded in the base pattern {(680189,T), (680209,A), (680290,C), (680314,G)} since all the pairs present in the former are also present in the latter pattern. The other base patterns, which are embedded in the base pattern {(680189,T), (680209,A), (680290,C), (680314,G)} and hence were removed from the list are as follows.

- {(680189,T), (680209,A)}
- {(680189,T), (680209,A), (680290,C)}
- {(680290,C), (680314,G)}
- {(680189,T), (680290,C), (680314,G)}
- {(680209,A), (680290,C), (680314,G)}
- {(680314,G)}
- {(680189,T), (680314,G)}
- {(680189,T)}
- {(680209,A)}
- {(680290,C)}

After removing embedded patterns, the following 7 base patterns remained. These were used for further processing.

| **Filtered *T. aestivum* Base Patterns** |
| --- |
| {(680189,T), (680209,A), (680290,C), (680314,G)} |
| {(680189,A), (680209,A), (680290,A), (680314,A)} |
| {(680189,T), (680209,G), (680290,C), (680314,G)} |
| {(680708,C), (680741,A), (680768,G), (680773,G)} |
| {(680708,T), (680741,A), (680768,T), (680773,C)} |
| {(680189,T), (680209,G), (680314,A)} |
| {(680708,C), (680741,G), (680768,G)} |

**Step 3: Assignment of base patterns to individual genomes**

The 7 base patterns that remained after removing potential sequencing errors and embedded base patterns were assigned to one or more genomes based on similarity with the diploids. The diploid bases at the HSP positions for the current gene are listed below.

| **HSP Position** | **HSP Bases** | ***T. urartu***  **(A diploid)** | ***Ae. speltoides***  **(B diploid)** | ***Ae. tauschii***  **(D diploid)** |
| --- | --- | --- | --- | --- |
| 680189 | A, T | T | T | T |
| 680209 | A, G | G | A | A |
| 680290^a^ | A, C | 0 | C | C |
| 680314 | A, G | A | A | G |
| 680708 | C, T | C | C | C |
| 680741 | A, G | G | A | A |
| 680768^b^ | G, T | * | T | T |
| 680773 | C, G | C | C | C |

^a^*T. urartu* has zero coverage at position 680290

^b^*T. urartu* has an ambiguous base (heterozygous base substitution) at position 680768

Using the above data, following similarity measures were calculated for the filtered base patterns.

| ***T. aestivum* Base Pattern** | ***T. urartu***  **(A diploid)** | ***Ae. speltoides***  **(B diploid)** | ***Ae. tauschii***  **(D diploid)** |
| --- | --- | --- | --- |
| {(680189,T), (680209,A), (680290,C), (680314,G)} | 33.3% | 75% | 100% |
| {(680189,A), (680209,A), (680290,A), (680314,A)} | 33.3% | 50% | 25% |
| {(680189,T), (680209,G), (680290,C), (680314,G)} | 66.6% | 50% | 75% |
| {(680708,C), (680741,A), (680768,G), (680773,G)} | 33.3% | 50% | 50% |
| {(680708,T), (680741,A), (680768,T), (680773,C)} | 33.3% | 75% | 75% |
| {(680189,T), (680209,G), (680314,A)} | 100% | 66.6% | 33.3% |
| {(680708,C), (680741,G), (680768,G)} | 100% | 33.3% | 33.3% |

Here, percentage identities lower than 50% are shown in red. When calculating the percentage identities, position 680290 was ignored when calculating the percentage identity of the base patterns containing this position (e.g. {(680189,T), (680209,A), (680290,C), (680314,G)} for *T. urartu* due to low coverage at this position. Similarly, the position 680768 was ignored from the base patterns containing it due to the presence of ambiguous base at this position when calculating the percentage identity for *T. urartu*.

The base pattern {(680189,A), (680209,A), (680290,A), (680314,A)} was forcibly assigned to B genome (shown in blue), which was designated as a distant genome (see above), due to low percentage identity with all other genomes.

**Step 4: Assignment of bases to individual genomes**

When assigning bases to individual subgenomes, the base patterns were first prioritized according to their percentage identities. This is shown below.

| **Subgenome** | ***T. aestivum* Base Pattern** | **Identity** |
| --- | --- | --- |
| A-subgenome | {(680189,T), (680209,G), (680314,A)} | 100% |
|  | {(680708,C), (680741,G), (680768,G)} | 100% |
|  | {(680189,T), (680209,G), (680290,C), (680314,G)} | 66.6% |
| B-subgenome | {(680189,A), (680209,A), (680290,A), (680314,A)} | 50% |
|  | {(680189,T), (680209,A), (680290,C), (680314,G)} | 75% |
|  | {(680708,T), (680741,A), (680768,T), (680773,C)} | 75% |
|  | {(680189,T), (680209,G), (680314,A)} | 66.6% |
|  | {(680189,T), (680209,G), (680290,C), (680314,G)} | 50% |
|  | {(680708,C), (680741,A), (680768,G), (680773,G)} | 50% |
| D-subgenome | {(680189,T), (680209,A), (680290,C), (680314,G)} | 100% |
|  | {(680189,T), (680209,G), (680290,C), (680314,G)} | 75% |
|  | {(680708,T), (680741,A), (680768,T), (680773,C)} | 75% |
|  | {(680708,C), (680741,A), (680768,G), (680773,G)} | 50% |

Note that the base pattern {(680189,A), (680209,A), (680290,A), (680314,A)}, which was forcibly assigned to the B-subgenome, is prioritized at the top (shown in blue).

Once bases were prioritized, the base assignments were made iteratively for each subgenome. For example, for D-subgenome the base assignments were first made using the pattern {(680189,T), (680209,A), (680290,C), (680314,G)} which had the highest percentage identity. In the second iteration, the base pattern {(680189,T), (680209,G), (680290,C), (680314,G)} was considered, which has the same bases at all HSP positions except 680209 where it has the base G instead of the base A. However, since the base pattern {(680189,T), (680209,A), (680290,C), (680314,G)} had a higher percentage identity with the diploid (100% as compared to 75%), base A was kept at position 680209. Moreover, as the pair (680209,G) now contradicted with the assignment made at this position, the base pattern {(680189,T), (680209,G), (680290,C), (680314,G)} was discarded and removed from the list of base patterns corresponding to D-subgenome. Other assignments were made in a similar fashion. The following bases were assigned to the three subgenomes.

| **HSP Position** | **HSP Bases** | **A-subgenome** | **B-subgenome** | **D-subgenome** |
| --- | --- | --- | --- | --- |
| 680189 | A, T | T | A | T |
| 680209 | A, G | G | A | A |
| 680290 | A, C | C | A | C |
| 680314 | A, G | A | A | G |
| 680708 | C, T | C | T | T |
| 680741 | A, G | G | A | A |
| 680768 | G, T | G | T | T |
| 680773 | C, G |  | C | C |

When making base assignments, the following base patterns were discarded as they contradicted with previously assigned bases.

| **Discarded Base Pattern** | **Reason** |
| --- | --- |
| {(680189,T), (680209,G), (680290,C), (680314,G)} | (680209,G) contradicted with the base ‘A’ assigned to this position in D subgenome |
| {(680708,C), (680741,A), (680768,G), (680773,G)} | (680708,C), (680768,G) and (680773,G) contradicted with bases assigned at these positions in B and D subgenomes |

**Step 5: Assignment of bases to individual genomes using discarded base patterns**

The two discarded base patterns shown above were re-assigned to subgenomes based on their percentage similarity with previously assigned bases.

| ***T. aestivum* Base Pattern**  **(discarded in Step 4)** | **A-subgenome** | **B-subgenome** | **D-subgenome** |
| --- | --- | --- | --- |
| {(680189,T), (680209,G), (680290,C), (680314,G)} | 75% | 0% | 75% |
| {(680708,C), (680741,A), (680768,G), (680773,G)} | 50% | 25% | 25% |

Identities less than 50% are again shown in red. The base pattern {(680189,T), (680209,G), (680290,C), (680314,G)} was assigned to the A- and D-subgenomes and the pattern {(680708,C), (680741,A), (680768,G), (680773,G)} was assigned to the A-subgenome.

Using the above percentage identities, base assignments were made as described in step 4. For the D-subgenome, no base assignments were made using the pattern {(680189,T), (680209,G), (680290,C), (680314,G)} as the base pattern {(680189,T), (680209,A), (680290,C), (680314,G)} which was used in step 4 to assign bases at these positions had a higher percentage identity with the diploid (100% compared to 75% for {(680189,T), (680209,G), (680290,C), (680314,G)}). Similarly, no base assignment was made for the A-subgenome using {(680189,T), (680209,G), (680290,C), (680314,G)}. However, the base G was assigned to the A-subgenome at position 680773, which was previously unassigned, using the base pattern {(680708,C), (680741,A), (680768,G), (680773,G)}. This resulted in the following final base assignments to the three subgenomes.

| **HSP Position** | **HSP Bases** | **A-subgenome** | **B-subgenome** | **D-subgenome** |
| --- | --- | --- | --- | --- |
| 680189 | A, T | T | A | T |
| 680209 | A, G | G | A | A |
| 680290 | A, C | C | A | C |
| 680314 | A, G | A | A | G |
| 680708 | C, T | C | T | T |
| 680741 | A, G | G | A | A |
| 680768 | G, T | G | T | T |
| 680773 | C, G | G | C | C |

The base assigned at position 680773 in step 5 is shown in blue.

# Note S2

# Base characterization using nullisomic-tetrasomic lines

Nullisomic-tetrasomic lines are a set of lines each missing a single chromosome (nullisomic), which is substituted by an additional copy of a homeologous chromosome (tetrasomic). For example, the line represented as N1AT1B lacks chromosome 1A but has two copies of chromosome 1B. Similarly, chromosome 1B is missing from the line called N1BT1D and is substituted by an additional copy of chromosome 1D.

The HSPs across six nullisomic-tetrasomic lines (for both chromosomes 1 and 5) were tabulated and positions where at least one genome had low coverage (< 3 reads) were ignored. The filtered lists were used for base assignment to the sub genomes as follows.

For each HSP position, a list of all bases present at that position was created. The base assignment was then done according to one of the following scenarios.

## Scenario 1 – All sub genomes contain different bases

In this scenario, the list of all bases present at the HSP position contains three distinct bases. A base was assigned to a particular genome when it was absent from the nullisomic lines that were null for the corresponding genome, but present in all the other nullisomic-tetrasomic lines.

Example:


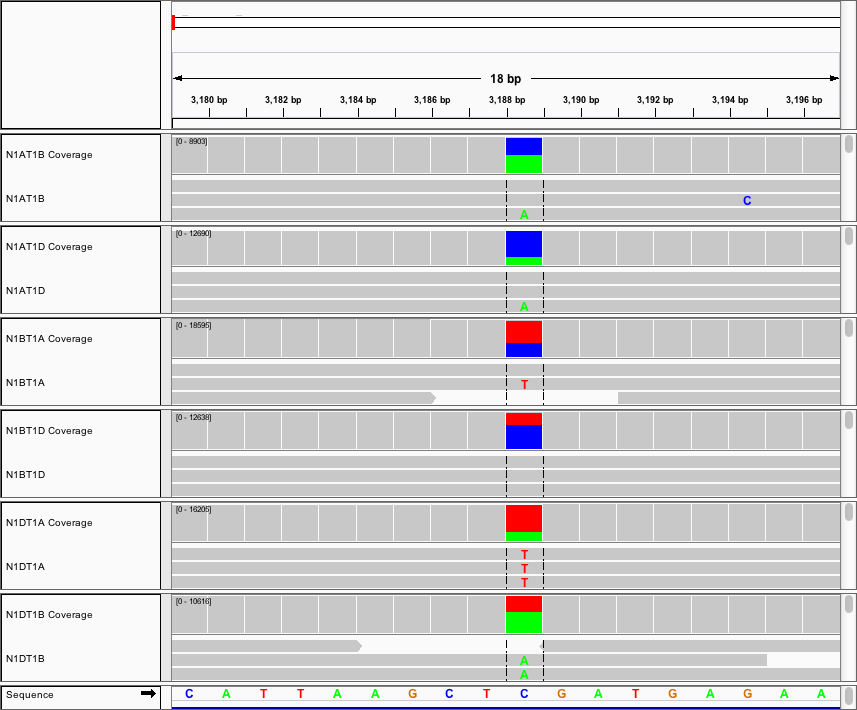


**Note:** The base A is shown in green, the base C is shown in blue and the base T is shown in red.

In the above example, there are three bases (A, C and T) at the HSP position. The base T is missing from the lines nullisomic for A subgenome (lines N1AT1B and N1AT1D) but is present in all other lines (lines N1BT1A and N1BT1D, and N1DT1A and N1DT1B). Similarly, bases A and C are missing from the lines that are nullisomic for B subgenome (lines N1BT1A and N1BT1D) and D subgenome (lines N1DT1A and N1DT1B) respectively, but present in the remaining lines. Consequently, the base T was assigned to *T. aestivum* A subgenome, the base A was assigned to *T. aestivum* B sub genome and the base C was assigned to *T. aestivum* D subgenome.

## Scenario 2 – Two sub genomes contain the same base

In this scenario, the list of all bases present at the HSP position contains two distinct bases. A base was assigned to a genome when it was absent from the nullisomic lines that were null for the corresponding genome, but present in all the other nullisomic-tetrasomic lines. Using this strategy, a base could be assigned to only one subgenome. The leftover base was assigned to the other two genomes.

Example:


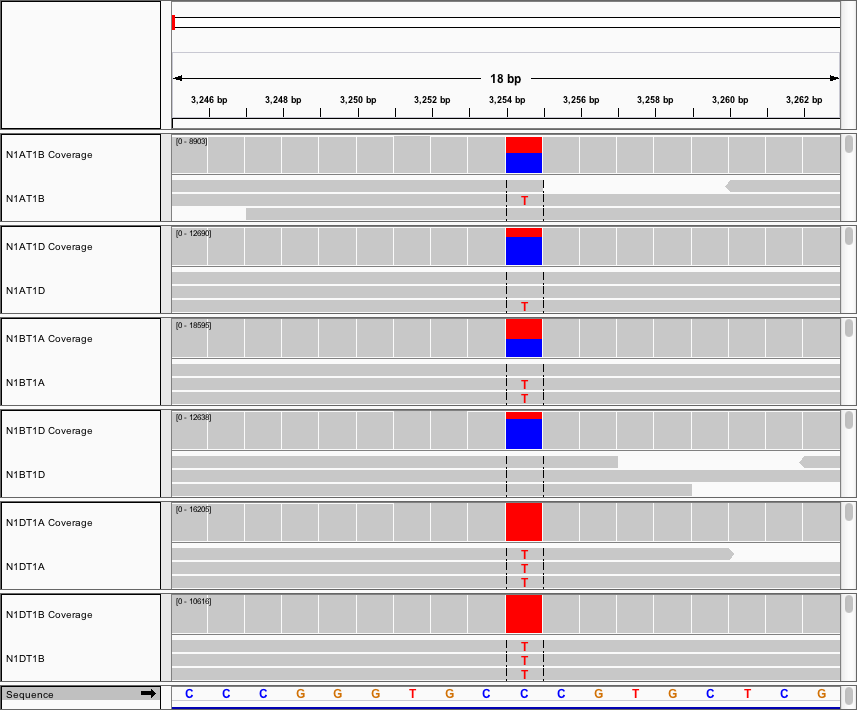


**Note:** The base C is shown in blue and the base T is shown in red.

In the above example, there are two bases (C and T) at the HSP position. The base C is missing from the lines that are nullisomic for D subgenome (lines N1DT1A and N1DT1B) but present in all other lines (lines N1AT1B and N1AT1D, and N1BT1A and N1BT1D). Consequently, C was assigned to *T. aestivum* D subgenome. The leftover base, i.e. T, was assigned to *T. aestivum* A subgenome and *T. aestivum* B subgenomes.

## Scenario 3 – One sub genome is silenced

In this scenario, the list of all bases present at the HSP position contains two distinct bases. A base was assigned to a genome when it was absent from the nullisomic lines that were null for the corresponding genome, but present in all the other nullisomic-tetrasomic lines. Using this strategy, a base could be assigned to two subgenomes. The subgenome for which no assignment could be made is defined as silenced at that position.

Example:


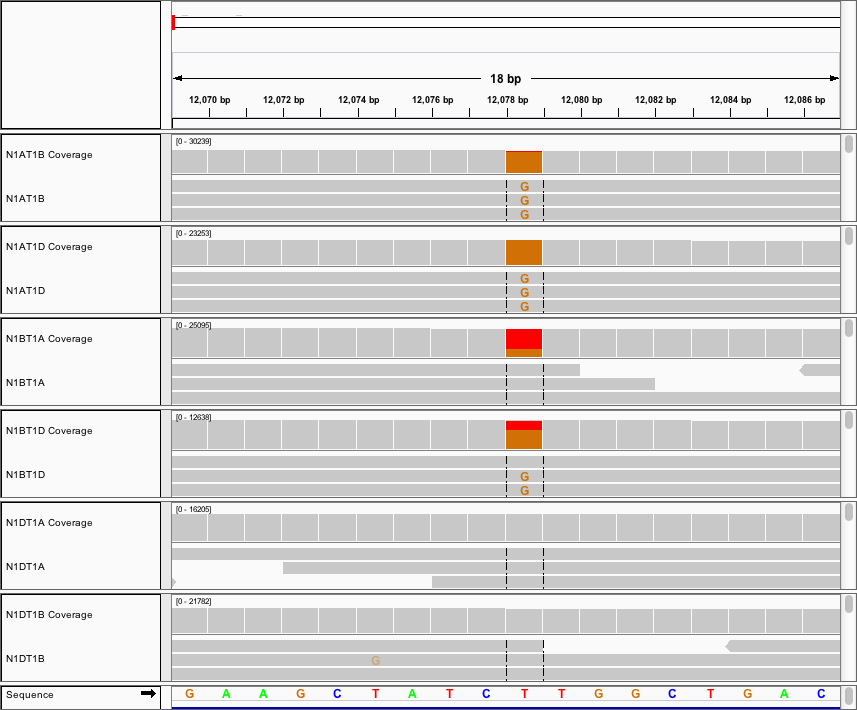


**Note:** The base T is shown in red and the base G is shown in brown.

In the above example, there are two distinct bases (G and T) at the HSP position. The base G is missing from the lines that are nullisomic for D subgenome (lines N1DT1A and N1DT1B) but present in all other lines lines (lines N1AT1B and N1AT1D, and N1BT1A and N1BT1D). Similarly, the base T is missing from the lines that are nullisomic for A subgenome (lines N1AT1B and N1AT1D) but present in all other lines (lines N1BT1A and N1BT1D, and N1DT1A and N1DT1B). Consequently, the base T was assigned to *T. aestivum* A subgenome and the base G was assigned to *T. aestivum* D subgenome. The *T. aestivum* subgenome B is silenced in this case.

## Note:

No assignment was made at positions where the nullisomic line for the same genome (for example, ‘nullisomic CS-A, tetrasomic CS-B’ and ‘nullisomic CS-A, tetrasomic CS-D’) did not agree, i.e. had different bases.

1. Winfield MO, Wilkinson PA, Allen AM, Barker GL, Coghill JA, Burridge A, Hall A, Brenchley RC, D'Amore R, Hall N et al. 2012. Targeted re-sequencing of the allohexaploid wheat exome. *Plant biotechnology journal* **10**(6): 733-742. [↑](#footnote-ref-2)
2. Li H, Durbin R. 2009. Fast and accurate short read alignment with Burrows-Wheeler transform. *Bioinformatics* **25**(14): 1754-1760. [↑](#footnote-ref-3)
3. Li H, Handsaker B, Wysoker A, Fennell T, Ruan J, Homer N, Marth G, Abecasis G, Durbin R. 2009. The Sequence Alignment/Map format and SAMtools. *Bioinformatics* **25**(16): 2078-2079. [↑](#footnote-ref-4)
